# Mechanistic of Vesicular Ethosomes and Elastic Liposomes on Permeation Profiles of Acyclovir across Artificial Membrane, Human Cultured EpiDerm, and Rat Skin: In Vitro-Ex Vivo Study

**DOI:** 10.3390/pharmaceutics15092189

**Published:** 2023-08-24

**Authors:** Afzal Hussain, Mohammad A. Altamimi, Obaid Afzal, Abdulmalik S. A. Altamimi, Mohhammad Ramzan, Tahir Khuroo

**Affiliations:** 1 Department of Pharmaceutics, College of Pharmacy, King Saud University, Riyadh 11451, Saudi Arabia; maltamimi@ksu.edu.sa; 2 Department of Pharmaceutical Chemistry, College of Pharmacy, Prince Sattam bin Abdulaziz University, Al-Kharj 11942, Saudi Arabia; o.akram@psau.edu.sa (O.A.); as.altamimi@psau.edu.sa (A.S.A.A.); 3 Department of Pharmaceutics, School of Pharmaceutical Sciences, Lovely Professional University, Jalandhar-Delhi GT Road, Phagwara 144411, Punjab, India; ramzan.pharma@gmail.com; 4 Department of Pharmaceutics, PGx Global Foundation, 5600 S Willow Dr Houston, Duarte, TX 77035, USA; tahir@pgxglobal.org

**Keywords:** acyclovir, permeation profiles, synthetic membrane, EpiDerm, rat skin, SEM, CLSM, AFM, in vivo TEWL study

## Abstract

Acyclovir (ACV) controls cutaneous herpes, genital herpes, herpes keratitis, varicella zoster, and chickenpox. From previously reported ACV formulations, we continued to explore the permeation behavior of the optimized ACV loaded optimized ethosome (ETHO2R) and elastic liposome (ELP3R) and their respective carbopol gels across artificial membrane, cultured human EpiDerm, and rat skin. Transepidermal water loss (TEWL), scanning electron microscopy (SEM), confocal laser scanning microscopy (CLSM), and atomic force microscopy (AFM) were used to investigate the mechanistic perspective of permeation behavior. The size values of reformulated ELP3-R and ETHO2-R were observed as 217 and 128 nm, respectively (close to previous report), whereas their respective gels showed as 231 and 252 nm, respectively. ETHO2R showed high elasticity, %EE, and low vesicle size. These were investigated for the diffusion rate of the drug permeation (3 h) across the artificial membrane, cultured human EpiDerm, and rat skin. ETHO2GR showed the highest permeation flux (78.42 µg/cm^2^/h), diffusion coefficient (8.24 × 10^−5^ cm^2^/h), and permeation coefficient (0.67 × 10^−3^ cm/h) of ACV across synthetic membrane, whereas diffusion coefficient (2.4 × 10^−4^ cm^2^/h) and permeation coefficient (0.8 × 10^−3^ cm/h) were maximum across EpiDerm for ETHO2GR. ETHO2R suspension showed maximized permeation flux (169.58 µg/cm^2^/h) and diffusion rate (0.293 mg/cm^2^/h^1/2^), suggesting the rapid internalization of vesicles with cultured skin cells at low viscosity. A similar observation was revealed using rat skin, wherein the permeation flux (182.42 µg/cm^2^/h), permeation coefficient (0.3 × 10^−2^ cm/h), and diffusion rate (0.315 mg/cm^2^/h^1/2^) of ETHO2R were relatively higher than ELP3R and ELP3GR. Relative small size (128 nm), low viscosity, ethanol-mediated ultra-deformability, high drug entrapment (98%), and elasticity (63.2) are associated with ETHO2R to provide remarkable permeation behavior across the three barriers. The value of TEWL for ETHO2R (21.9 g/m^2^h) was 3.71 times higher than untreated control (5.9 g/m^2^h), indicating ethanol-mediated maximized surficial skin lipid perturbation at 3 h of application, whereas the respective ETHO2GR-treated rat skin had TEWL value (18.6 g/m^2^h) slightly lower than ETHO2R due to gel-based hydration into the skin. SEL, CLSM, and AFM provided a mechanistic perspective of ETHO2R and ELP3R-mediated permeation across rat skin and carrier-mediated visualization (skin–vesicle interaction). AFM provided detailed nanoscale surface roughness topographical parameters of treated and untreated rat skin as supportive data to SEM and CLSM. Thus, ethosomes ETHO2R and respective gel assisted maximum permeation of ACV across rat skin and cultured human EpiDerm to control cutaneous herpes infection and herpes keratitis.

## 1. Introduction

A report was published (in 2020) by the World Health Organization (WHO) and collaborative partners wherein 5% of the world population (187 million people) suffered from genital herpes ulcer due to herpes infection (commonly by HSP-2) [1]. The genital herpes infection was associated with HIV, recurrence nature of infection, oral sexual performance, and other complications (psychological distress, cold sores, and pain). A case of disseminated cutaneous HSV-1 (33-year-old male patient) was observed as the first manifestation of human HIV (polymerase chain reaction confirmed HSV-1), characterized by painful cutaneous vesicular rashes (covering about 90% of his body surface area), temporal lobe encephalitis, and multiple organ failure [2]. Acyclovir sodium is potentially effective against cutaneous herpes, genital herpes, varicella zoster, chicken pox, and herpes keratitis. However, oral delivery is clinically limited due to poor bioavailability (10–30%), high dose (200 mg five times a day for 10 days), and dosing frequency (half-life = 3.25 h). The drug is associated with several side effects due to high oral dose (headache, rashes, and diarrhea) and route administration (bolus injection causes renal precipitation) [3]. To circumvent the above issues and sustained delivery of the drug, a transdermal route of administration was adopted, and various literatures suggested convincing findings for improved drug permeation and in vivo performance using novel carrier systems, such as vesicular systems (liposomes, elastic liposomes, and ethosomes), nanoemulsion, and hydrogel microneedles with or without gel. Jain et al. [3] formulated elastic liposomes and compared flux profiles against plain drug solution and conventional liposomes. Elastic liposomes showed flux values as 6.3 and 2 times higher than plain drug solution and liposomes, respectively. Moreover, plasma drug concentration reached 105 ng/mL (4.2 folds higher than liposomes) at 24 h after transdermal delivery in a rat model. Considering therapeutic effectiveness, the achieved flux value (6.21 µg/cm^2^/h) was found to be low [3], and physical stability at varied conditions was not explored. The drug loaded in carbopol gel matrix containing permeation enhancers (d-limonene, dimethyl sulfoxide, and sodium taurodeoxycholate) were tailored to study its effectiveness against cutaneous herpes simplex virus. These authors investigated that ex vivo permeation parameters (flux, permeation coefficient, and enhancement ratio) and the drug deposition using carbopol (containing permeation enhancers as described before) within rat skin were relatively higher than conventional formulation [4]. In another strategy, Donnelly et al. firstly attempted to describe hydrogel-forming microneedle arrays using super-swelling polymeric aqueous components (20% *w*/*w* Gantrez S-97, 7.5% *w*/*w* PEG 10,000 and 3% sodium carbonate) and 37% of the loaded drug (ibuprofen) was delivered at 24 h [5]. Based on these reported findings, permeation enhancer, carbopol gel, and microneedle array-based formulations provided quite convincing outcomes, such as high permeation parameters and maximized drug deposition. A chinse natural permeation enhancer “oleanolic acid” was reported to be used (0.05–20% *w*/*w*) for maximizing transdermal delivery of the drug (0.01–30% *w*/*w*) for access to the dermal region [6]. Almehmady and Ali reported a garlic oil-based self-nanoemulsifying drug delivery system (SNEDDS) for ACV delivery using transdermal film. A blend of tween 20-span 20 and propylene glycol served as surfactant and co-surfactant, respectively. The optimized formulation was comprised of 10.4% of the oil, 65% of the surfactant blend, 24.8% of PG, and 200 mg of ACV. It is clear from the reported values that SNEDDS requires a huge quantity of surfactants with chances of toxicity and irritation. Therefore, authors investigated transepidermal water loss (TEWL) and skin irritation in an animal model [7]. Recently, Shekh et al. reported an oxidized chitosan-modified electrospun scaffold with well biocompatibility and controlled release of ACV after transdermal application. However, the authors primarily investigated in vitro assessment of the electrospun constructs and hemo-compatibility. Ex vivo and in vivo parameters for topical or transdermal application had not been studied in any animal models [8]. In further development, nanofibrous core-sheet-based electrospun was explored for topical delivery of two drugs to treat herpes labialis. The authors used ACV and dexpanthenol as the core and the sheet, respectively, in the nanoelectrospun fibrous patch. Both drugs were completely released within 2000 s and the drug-loaded HPLC nanofiber showed excellent activity against herpes simplex virus. However, the prepared product had relatively low potential performance compared to commercial Zovirax cream (5% *w*/*w*) [9]. Therefore, a double blind randomized clinical trial was conducted to compare the therapeutic efficacy of ACV cream and ACV-loaded nanofiber patch to treat recurrent labia herpes (60 patients in three groups) [10]. The product was not substantially effective to shorten crusting time and healing time among all groups tested.

However, preclinical data seem to be overestimated when correlated for clinical efficacy under the same experimental condition and composition. Therefore, we had formulated binary ethosomes and elastic liposomes for the transdermal delivery of ACV salt (sodium salt of ACV based on the data gleaned from the literature, properties of the drug, patient-related issues with conventional dosage form (parenteral and oral), and safety concern of formulation). Formulations were evaluated for in vitro, ex vivo, and in vivo parameters for completing the proof of concept. In our previous study, a comparative assessment was performed and highlighted TEWL, hemocompatibility, ex vivo permeation profile in rat model, and Draize test. ELP3 and ETHO2 were two optimized elastic liposomes and ethosomes formulations, respectively. Both were optimized based on small vesicle size, high entrapment efficiency, maximum steady state permeation flux (*J*_ss_ = 87.6 ± 4.8 μg/cm^2^/h and enhancement ratio = 7.3 for ETHO2 and *J*_ss_ = 12 ± 2.6 μg/cm^2^/h for drug solution), drug deposition, high deformability, elasticity behavior, and in vitro drug release in phosphate buffer [11]. In the present study, we attempted to fill the research gap of our previous investigation. Therefore, we elaborated mechanistic perspective of permeation of both ELP3 and ETHO2 with and without carbopol gel using three different barriers, such as artificial membrane, rat skin, and cultured human EpiDerm. Scanning electron microscopy (SEM), confocal laser scanning microscopy (CLSM), and atomic force microscopy (AFM) were used to inspect and examine microscopic changes after treatment (instrumental-based evidence). The study provided a detailed background of permeation parameters and a mechanistic perspective of permeation across rat skin, followed by a comparison with untreated skin.

## 2. Materials and Methods

### 2.1. Materials

Acyclovir (ACV, 99.8% pure) was received as a gift from Cipla Pharmaceuticals (Mumbai, Maharashtra, India). Ethanol, methanol, and acetonitrile were HPLC (high performance liquid chromatography) grade, and these were purchased from Merck, Mumbai, Maharashtra, India. Buffer reagents (potassium dihydrogen phosphate, sodium chloride, and sodium hydroxide) were procured from S.D. Fine Mumbai, India. Carbopol 934 was purchased from Thane, Maharashtra, India. Rhodamine 123 was procured from Merck, Mumbai, India. Cholesterol (extra pure) was purchased from Sigma Aldrich, St. Louis, MO, USA. Phosphatidylcholine (PC) was purchased from Ludwinshafen, Germany. Propylene glycol (PG), tween 80, and span 80 were procured from Himedia Chemicals (Mumbai, India). In-house, installed distilled water was used as an aqueous solvent. For HPLC mobile phase preparation, Milli-Q water was used (Millipore, Billerica, MA, USA).

### 2.2. Methods

#### 2.2.1. Vesicular Formulation Containing ACV Probed with Rhodamine 123

In this study, we selected various vesicular formulations from our previously published reports [11,12]. In brief, ELP3, ELP3-gel, ETHO2, and ETHO2-gel were reformulated with rhodamine-123 (0.1%). The dye solution was prepared in distilled water with a final concentration of 0.1% *w*/*v*. A summary of composition of formulations has been compiled in Table 1. The dye solution worked as control. Briefly, a precise amount of PC (phosphatidylcholine, 850 mg) and span 80 (150 mg) were completely dissolved in the organic solvent mixture (chloroform: methanol = 2:1, 2 mL). The obtained solution was transferred into a round bottom flask (previously cleaned and dried) for developing film. A thin film was developed using a rotary evaporator. The completely dried film (free from the organic solvent) was hydrated using 10 mL of phosphate buffer solution containing the dye and the drug (100 mg) (pH 7.4). The obtained milky colloidal ELP3 was stored at low temperature overnight for the activation of vesicles.

A similar method was adopted for ethosomes. ETHO2 were formulated using PC (700 mg/mL), cholesterol (9.65 mg/mL), and tween 80 (25 mg/mL) as per the method reported before [11]. In the hydration step, ETHO3 was of a different composition. ETHO2 was hydrated with the buffer containing ACV, the dye, and a mixed blend of propylene glycol-ethanol (8% in 0.6 ratio) [11]. Organic phase generated a thin film inside the round bottom flask using a rotary evaporator. The completely dried film was hydrated with hydration fluid and vesicular systems were prepared at 50–55 °C. Both formulations were sealed and placed in a refrigerator to activate vesicle overnight. Following that, the colloidal suspension was subjected for probe sonication (Sonoplus™ HD; Bandelin Electronics, Berlin, Germany) for 4 min to reduce size [11]. Each formulation was freeze-dried (Heto PowerDry LL 1500, Freeze Dryer, Denmark) for incorporation into a gel carrier.

ELP3-gel-R and ETHO2-gel-R were prepared using 1% *w*/*w* carbopol gel. For this, a weighed amount of carbopol 934 was dispersed in a warm distilled water to obtain a final strength of 1% *w*/*w*. The dispersed gel was vigorously stirred with a mixer at high speed (10,000 rpm). The obtained gel was treated with a few drops (3–5 drops) of triethanolamine (base) as a cross-linking agent. The acidic solution of carbopol dispersion was triggered for cross-linking under triethanolamine and became a transparent viscous gel. An equal weight of gel and lyophilized formulation was mixed together using a homogenizer to obtain a consistent gel. The final concentration of ACV in gel product was approximately 5% *w*/*w*. The final pH of each formulation was adjusted to 6.8–7.1 to obtain a good consistency.

#### 2.2.2. Characterizations

ETHO2R, ELP3R, ETHO2-GR, and ELP3-GR were characterized for vesicle size, size distribution, and zeta potential using Malvern zetasizer (Malvern zetasizer, Westborough, MA, United State of America). For morphology and surface topographical assessment, an atomic force microscopy (AFM) (Solver Pro 47, Saint Petersburg, Russia) study was used for each ELP3R and ETHO2R at room temperature, a scanning rate of 0.8 Hz, and a semi-contact angle [13]. The elasticity of ETHO2R and ELP2R was determined using a membrane filter method wherein each sample was passed through the membrane and size was measured using a zetasizer [14]. The value of pH was measured using Hanna digital pH meter. Viscosity was measured using cone-plate viscometer (Bohlin Visco-88, Malvern, UK) at room temperature (25 ± 1 °C). Percent entrapment efficiency (%EE) was estimated using an ultracentrifugation method. The sample was initially filtered through a membrane filter (0.2 µm) to obtain only vesicles. The undissolved drug was retained. Following that, each sample (5 mL) was taken into a centrifugation plastic tube and subjected to centrifugation at 18,000 rpm to settle the vesicles at the bottom. The settled pellet was carefully collected and dissolved in methanol to obtain the entrapped drug. This was further filtered, and the filtrate was used to estimate the %EE following = Equation (1). The supernatant was also used for back calculation and free drug estimation. Experiments were carried out in triplicate to obtain mean and standard deviation.

% EE = [(Amount of the drug trapped)/(Initial drug content)] × 100(1)

#### 2.2.3. In Vitro Drug Permeation across Artificial Membrane

A cellulose acetate membrane (0.45 µm) was used as an artificial membrane to determine the drug release employing Franz diffusion cell (Electro lab, Evansville, IN, USA). The membrane was not wetted to avoid any interference in results. Both chambers of the diffusion cell were assembled in such a way that the receptor chamber was filled with phosphate buffer solution (pH = 7.4). The membrane was placed between the chambers. The receptor chamber was regularly stirred using an inert Teflon coated rice bead over a magnetic stirrer. The donor chamber received the formulation (0.5 mg contained in loaded dose of ELP3-R, ETHO2-R, ELP3-gel-R, and ETHO2-gel-R). The temperature was maintained using circulating heated water in the jacket of the chamber. The temperature and stirring were kept constant throughout the study for each formulation [11]. Sampling (1 mL) was performed at predetermined time points (30, 60, 120, 150, and 180 min). The withdrawn volume was replaced with fresh release medium to maintain sink condition. The content of the drug released was assayed using HPLC at 254 nm, as reported before [11]. The study was replicated for the mean and standard deviation (*n* = 3).

#### 2.2.4. In Vitro Drug Permeation across Human Culture EpiDerm Membrane

EpiDerm (cultured human epidermal keratinocytes) was used as a viable alternative to human skin, as it is similar as human keratinocytes in term of structure (multi-layered basement membrane and in vivo such as lipid profile) and functionality and explored dermal toxicity and irritability [15]. The cultured skin was mounted between both chambers and the study was conducted under similar experimental conditions, as mentioned in Section 2.2.3. The sampling time (30–180 min), replacement volume (1 mL), and dose (0.5 mg) were kept constant. The drug was quantified using a HPLC method for each formulation (ELP3-R, ETHO2-R, ELP3-gel-R, and ETHO2-gel-R). Permeation parameters were calculated, specifically, the permeation coefficient, percent cumulative amount of drug release, permeation flux, and diffusion coefficient. Topically applied product is the best studied using cultured human epidermal keratinocytes (EpiDerm), which is as similar to human skin as possible [16]. This is a suitable model for topical products simulating the human nature of skin and was pre-treated PBS (pH 7.4) for 20 s to facilitate the outer layer for diffusion. The jacketed water was circulated around the receptor chamber-maintained temperature (32 ± 1 °C) throughout the study. The effective surface area for passive diffusion of the drug was 1.34 cm^2^ functional at 32 ± 1 °C [16].

#### 2.2.5. Ex Vivo Drug Permeation and Drug Deposition Using Rat Skin

Ex vivo drug permeation was carried out using abdominal rat skin excised from ethically sacrificed rat. Healthy animals (weighing about 300 g of both sex) were issued and approved from the Institutional Ethical Committee (approval number PCTE/LDH/1370/2013) of Punjab, (PCTE Institute, PTU, Ludhiana, Punjab, India), as reported in a previous study [11]. Cleaned (free from fatty debris and hairs) and healthy skin was placed between both the chambers of Franz diffusion cells. The epidermal surface was on the dose-loading side, whereas the dermal surface faced the drug release medium. A constant amount of the dose (equivalent to 0.5 mg) was loaded onto the epidermal side of donor chamber. The receptor chamber filled with release medium was constantly stirred under a magnetic stirrer previously set at a fixed temperature and rotation. The medium was stirred using rice beads. Sampling volume (1 mL), sampling time points (30–180 min), release medium (PBS), and replacement volume (1 mL) were fixed as before. The drug was quantified using HPLC at 254 nm. The study was conducted up to 180 min to avoid the loss of the natural integrity of tissue and tissue viability [17]. The study was replicated to obtain mean value.

#### 2.2.6. In Vivo Transepidermal Water Loss (TEWL) Study Using Rat Skin

The study was conducted using healthy rats. Animals were randomly divided into five groups. These are designated as A, B, C, D, and E. Group A, B, C, and D received ELP3-R, ETHO2-R, ELP3-gel-R, and ETHO2-gel-R, respectively. Group E served as control (untreated). Each group contains three rats. Rats were quarantined for 24 h and housed in AC condition with sufficient access to water. Following that, the dorsal portion of each rat was used to make a circular area of 1 cm^2^ free from hairs. The area was properly marked and labelled for the sample application. The circular area was applied with the respective formulation (equivalent dose). After 3 h of application, the treated area was cleaned using non-adhesive tissue paper. Animals were neither sacrificed nor anaesthetized. Wet tissue paper was avoided to interfere the result. The probe of TEWL was placed (perpendicular to the surface) on the spotted area and stabilized in 60 s. TEWL was measured at 0 time point (before treatment) and after 180 min of treatment. The values were expressed in g/h.m^2^ and compared with untreated control [18].

#### 2.2.7. Confocal Laser Scanning Microscopy (CLSM)

After completion of ex vivo permeation study using rat skin, the skin portion (effective functional surface area) was removed, and the remaining formulation was washed from the treated surface using distilled water. Four samples (ELP3-R, ETHO2-R, ELP3-gel-R, and ETHO2-gel-R) of the treated skin and the untreated skin were sliced into small pieces (0.2 cm × 0.5 cm) using a cryostat microtome (CM 1900, Leica, Germany). The tissue specimen (a thickness of 25 µm) was placed on the glass coverslip and air-dried for 12 h [19]. Each tissue was visualized under CLSM (Zessi LSM 710, Jena, Germany) and evaluated for vesicle penetration across skin by visualization with camera (Fluorescence Correlation Microscope-Olympus FluoView FV1000, Olympus, Melville, NY, USA) coupled with an argon laser beam with excitation at 488 nm and emission at 590 nm [20,21].

#### 2.2.8. Vesicle–Skin Interaction Study Using Scanning Electron Microscopy (SEM)

The skin tissue specimens obtained from an ex vivo study were used to visualize under SEM. The technique helped to examine microscopic changes in the treated skin surface and the permeation of vesicle across skin. The skin was excised from an ex vivo study, removed, and then the adhered sample was removed using running water. The specimen sample was transferred to formalin solution to preserve the structural changes by dehydration. The tissue was sliced into small pieces and placed on the glass cover slip. Each tissue sample was air-dried overnight followed by drying under oven at 37 °C for 12 h. The tissue was placed on the copper grid using double adhesive tape. The epidermal portion was facing air and the surface was coated with gold. The coated sample was loaded in the SEM (Carl Zeiss, EVO43, SEM, Jena, Germany) for visualization at varied resolution and magnification. The sample was scanned at various regions of the skin to examine structural changes and vesicle interaction with skin.

#### 2.2.9. Topographical Study Using AFM Technique

The principle of AFM technique is based on the scanning ability of sharp-tipped cantilever (prime component) for the sample surface in the x-y plane [22]. An atomic force microscopy study was used to investigate the nanoscale surface topographical variation of the treated rat skin as compared to untreated skin. After completion of the CLSM study, the sample specimens were used to assess topographical parameters such as mean roughness (Ra in nm), roughness skewness, Kurtosis, enthalpy, and root mean square roughness (rms in nm). For this, the sample was placed on a glass cover slip previously coated with poly-L-lysin (tissue fixative) to withstand a sharp-tipped cantilever-based exerted pressure on the sample. The sample was completely air-dried for 24 h at room temperature (32 °C). Each sample was placed in such a way that the epidermis faced air on the slide. The technique was applied to investigate and correlate the mechanical properties of cornified cells of SC with integrity and barrier function in untreated and treated skin. The glass slide with specimen was properly placed on the plate-form and against cantilever. The scanning process was conducted in semicontact mode and phase contrast imaging. Images were obtained using Solver P47 microscope (Russia) at a spring constant of 11.8 N/m, fixed radius of curvature, and a resonance frequency of 240 kHz [23]. Images were analyzed using Nova software (AFM Solver Pro 47 (NT MDT, Saint-Petersburg, Russia).

#### 2.2.10. Data Analysis for the Permeation Profile

To calculate permeation parameters across rat skin, EpiDerm, and the membrane, various mathematical models were employed, as shown below. Various plots of the cumulative amount of ACV permeated versus time (using Fick’s first law) or the square of time (to calculate the diffusion coefficient using Higuchi model). The estimated flux (*J*) was determined using the following Equation (2) of Fick’s first law:

*J* = (*dQ*)/*Adt* = *D* × (*dC*/*dx*) = *D* × (C_1_ − C_2_)/*x* (2)

where *J*, *Q*, *A*, *D*, and *x* are the permeation flux (µg/cm^2^/h), loaded ACV quantity, effective functional area for passive diffusion, the diffusion coefficient, and membrane thickness (cm). P is the permeation coefficient (cm/h), whereas C_1_–C_2_ is designated as the concentration gradient. The dQ/dt was the fitted permeation of ACV against time. The value of “P” is estimated as a permeation velocity of the drug permeated through EpiDerm using the slope of percent (%) drug permeated versus time profile as:

P = (m × V)/*A* (3)

where m, V, and A are the slope, volume of receptor chamber of Franz diffusion cell, and operational surface area available for permeation. The diffusion coefficient (*D*) (diffusivity) was calculated by plotting the content of ACV diffused per unit area (µg/cm^2^) against the square root of time (h) using Equation (4):

(4) $$D=\frac{J}{\left(\frac{dC}{dx}\right)}$$

where *J* and “*dC*/*dx*” are the flux and the concentration difference (C_1_–C_2_) diffused across *dx* (0.2 cm as the thickness of EpiDerm), respectively [24,25,26].

## 3. Results and Discussion

### 3.1. Various Reformulated Products of ACV Probed with Rhodamine 123

The composition of various R123-probed binary ethosomes and elastic liposomes is summarized in Table 1. It is clearly mentioned that ELP3-R, ETHO2-R, ELP3-gel-R, and ETHO2-gel-R were evaluated for prime parameters (vesicular size, shape, percent entrapment, elasticity, final pH, and viscosity). The vesicle size of ELP3-R and ETHO2-R were observed as 217 and 128 nm, respectively (close to previous report), whereas their respective gels showed as 231 and 252 nm, respectively. The slight increase in vesicle size may be correlated to hydrogel causing swelling but insignificant (*p* ˃ 0.05). A similar pattern of intrinsic changes was observed for elasticity, %EE, and viscosity. The gel viscosity is significantly higher than the respective vesicle colloidal suspension. A considerable difference in pH was due to the acidic nature of carbopol polymeric hydrogel. The pH value of hydrogel was kept below neutral (pH ˂ 7.0) for desired consistency and viscosity. The hydrogel exhibits maximum solubility under slight acidic pH due to pH-mediated facilitated cross-linking. An insignificant difference in these evaluated parameters may be due to approximately similar composition and vesicular system. The developed ELP3 (elastic liposomes containing 7% ethanol in hydration medium) and ETHO2 as binary ethosomes (containing 45% ethanol in hydration medium) were of a similar composition, except dye (0.1%). The addition of probed R123 could not change significant characteristics of these explored vesicular systems. Therefore, we aimed to investigate diffusion behavior, permeation profile, and the degree of the drug penetration across rat skin. The permeation coefficient across EpiDerm may be valuable for simulating preclinical finding to predict in vivo performance in human skin. ELP3R, and ETHO2R are colloidal suspension whereas their respective gel (by swelling and compensating trans-epidermal water loss) may benefit permeation profiles and diffusion coefficient across EpiDerm, and rat skin. A comparison was established among them for permeation profiles in the subsequent section.

**Figure 1:**
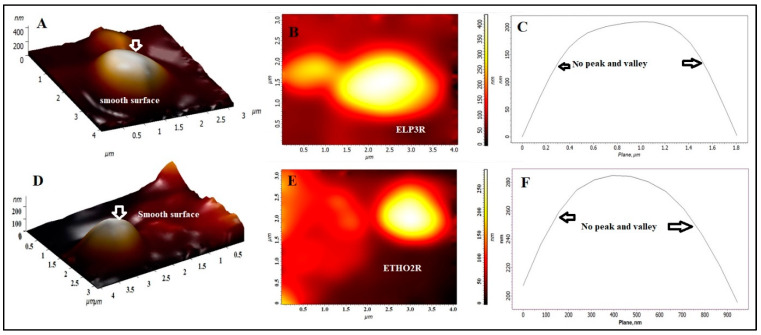
Atomic force microscopy for morphology and topographical analysis. (**A**) Three-dimensional image of ELP3R exhibiting smooth surface and spherical vesicle, (**B**) two-dimensional image of ELP3R revealing surface margin and layering, (**C**) surface roughness curve parameter of ELP3R wherein the absence of peaks and valley indicated smooth behavior of ELP3R vesicles, (**D**) three-dimensional image of ETHO2R exhibiting smooth surface and spherical vesicle, (**E**) two-dimensional image of ETHO2R revealing surface margin and layering, (**F**) surface roughness curve parameter of ETHO2R wherein the absence of peaks and valley indicated smooth behavior of ETHO2R vesicles (relatively smaller than ELP3R).

To investigate nanoscale surface topographical properties of ELP3R and ETHO2R vesicles, AFM was a suitable tool, and the result is portrayed in Figure 1A–F. Three-dimensional and two-dimensional images of ELP3R are elicited in Figure 1A,B, respectively. The vesicle of ELP3R from the targeted area of the specimen is spherical and smooth in topological property. The roughness curve showed no bunch of peaks and valleys, as shown in Figure 1C. A similar observation was inspected in ETHO2R, except for size. In terms of surface roughness parameters, both vesicular formulations were smooth and firm in architecture. However, ETHO2R exhibited relatively small vesicle size as compared to ELP3R, as shown in Figure 1D,E. The roughness curve was approximately the same as that observed in ELP3R (Figure 1F). The values of average roughness, root-mean-square (rms), surface skewness, and enthalpy for ELP3R were estimated as 5.2 nm, 50.12 nm, 0.409, and 5.4, respectively. For ETHO2R, these values were obtained as 4.4 nm, 44.14 nm, 1.3, and 10.07, respectively. Thus, the surface roughness parameters (Ra and rms) were quite close to each other, suggesting a smooth surface of ELP3R and ETHO2R. This finding can be further correlated with the surface roughness curve obtained from the fixed targeted zone of analysis.

### 3.2. In Vitro Drug Permeation across Artificial Membrane

**Figure 2:**
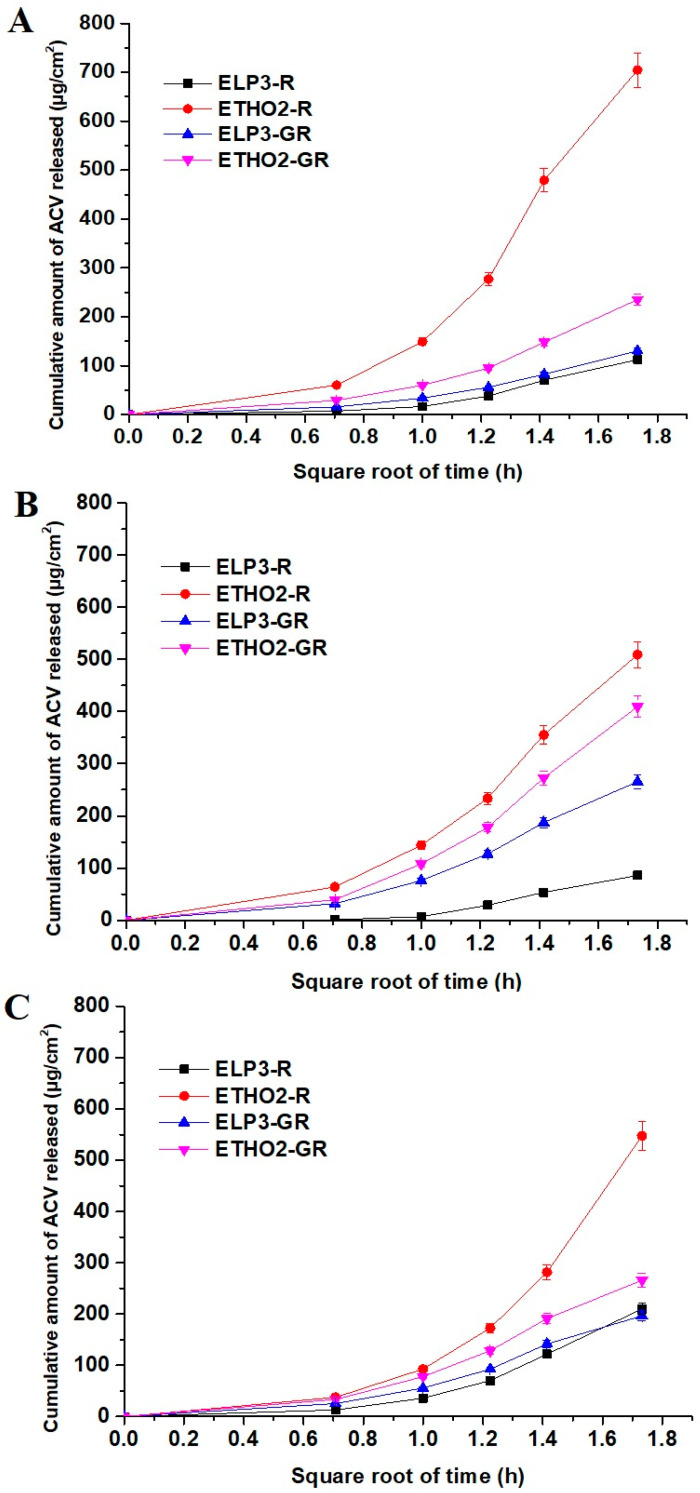
(**A**) Diffusion profile (diffusion coefficient) of ACV versus the square root of time (h) from the developed formulations through synthetic membrane, (**B**) diffusion profile of ACV through EpiDerm, and (**C**) diffusion profile of ACV through rat abdominal skin at 32 °C temperature (mean ± standard deviation, *n* = 3 and *p* < 0.05).

The results are illustrated in Figure 2A and Table 2. The permeation rate of ETHO2R and ETHO2GR exhibited relatively higher values than ELP3R and ELP3GR, as shown in Figure 2A. The diffusion coefficient values (D) of ETHO2R and ETHO2GR were found to be 7.57 × 10^−5^ cm^2^/h and 8.24 × 10^−5^ cm^2^/h, respectively, whereas these values were observed as 7.98 × 10^−6^ cm^2^/h and 2.54 × 10^−5^ cm^2^/h for ELP3R and ELP3GR, respectively. It is quite clear that ethosomes ETHO2R and ETHO2GR caused facilitated permeation across artificial membrane, which may be attributed to small vesicle size as compared to ELP3R. Secondly, ethosomes are ethanolic rich vesicular system for ultra-deformability and elasticity across minute membrane pores size [11]. Similarly, flux, permeation coefficient, and diffusion rate were higher for ethosomes (ETHO2R) as compared to elastic lipoosmes (ELP3R), as shown in Figure 2A and Table 2. ELP3R and ELP3GR revealed diffusion rate values as 0.0648 and 0.0752 mg/cm^2^/h^1/2^, respectively, which may be related to relatively larger vesicles size as compared to ethosomes (ETHO2R) [11]. Thus, vesicle size, the elastic nature of lipid bilayer, and ethanolic content played together to control diffusion rate, diffusion coefficient, permeation coefficient, and flux studied over a period of 3 h.

### 3.3. In Vitro Permeation Assessment Using EpiDerm

The developed four formulations were investigated for permeation and diffusion parameters. The result is illustrated in Table 2. The cultured human EpiDerm is the most differentiated model and serves as a viable substitute to study diffusion and permeation coefficients so that we can simulate in vivo performance [16]. Figure 2B elicited the pattern of cumulative drug permeation across cultured human EpiDerm versus square root of time (h). It is clear that ethosomes containing a relatively high content of ethanol facilitated permeation compared to other formulations such as elastic liposomes (ELP3-R and its gel). The values of permeation flux across human-cultured EpiDerm were found to be 169.58, 136.45, 28.61, and 88.55 µg/cm^2^/h for ETHO2R, ETHO2-GR, ELP3R, and ELP3-GR, respectively. The permeability coefficients of ETHO2R (0.63 × 10^−3^ cm/h) and ETHO2-GR (0.80 × 10^−3^ cm/h) were found to be quite high as compared to ELP3R (0.25 × 10^−3^ cm/h) and ELP3-GR (0.36 × 10^−3^ cm/h). These permeation parameters suggested that vesicular-based transdermal delivery of ACV is convincing for effective therapeutic benefit, particularly ethosomes. This might be due to ethanol serving as edge activator and membrane plasticizer for augmented ultra-deformability and significant flexibility. These concerted properties assist vesicles to be squeezed into deeper dermal side across tiny microscopic pores and interstitial spaces. Moreover, ethanol causes reversible changes in skin lipid and architecture for maximized drug flux and permeation across stratum corneum. The values of the diffusion coefficient (*D*) (Table 1) for ETHO2R, ETHO2-GR, ELP3R, and ELP3-GR suggested that gel provided a relatively high diffusion coefficient due to a high drug diffusion rate as compared to respective ELP3R and ETHO2R. This may be prudent to correlate with the hydration provided to cultured EpiDerm for ease in the drug permeation and diffusion. Carbopol is hydrophilic and bio-adhesive polymeric gel is capable of loading a high water content (high swelling index). Thus, the high diffusion coefficient of ELP3-GR and ETHO2-GR may be attributed to the high diffusion rate, gel-based hydration, and extended bio-adhesiveness to the surface of EpiDerm to develop a high concentration gradient released from ethosomes at skin pH [16]. Notably, ELP3R revealed the lowest value of cumulative amount of drug permeation as compared to others, which may be correlated to the low content of ethanol (7% *v*/*v* in EP3R) and poor hydration (as shown in Figure 2B).

### 3.4. Ex Vivo Permeation of ACV across Rat Skin

Considering rat skin, a common ex vivo model for permeation parameters was used. These profiles were compared with the results obtained from artificial membrane and cultured human EpiDerm. The result is summarized in Table 2 wherein permeation rate, permeation coefficient, and the drug diffusion coefficient values can be correlated to understand the impact of composition, gel, and hydration. The permeation flux values of the drug across rat skin were found to be 182.42, 88.64, 70.03, and 65.55 µg/cm^2^/h for ETHO2R, ETHO2-GR, ELP3R, and ELP3-GR, respectively. Notably, gel caused mitigated flux due to the viscous nature of gel in both cases, whereas colloidal suspension of elastic liposomes and ethosomes resulted in rapid flux due to the direct interaction of vesicles with skin components. A similar trend was observed for diffusion coefficient, permeation coefficient, and diffusion rate for an obvious reason, as shown in Table 1 and Figure 1C. Comparing the flux values of ETHO2R and ETHO2-GR, ETHO2R showed the highest flux wherein vesicles fragmentation is thus appreciable only if permeation flux is remarkable. In the case of gel, permeation rate and flux values were found to be low, which may be correlated with the fact that least or no vesicle size diminution occurs at low barrier passage rate [27]. The shape of permeation profile is approximately linear with the square root of time and the drug release of 210.1 µg/cm^2^, 196.66 µg/cm^2^, and 265.9 µg/cm^2^ for ELP3R, ELP3-GR, and ETHO2-GR, respectively, at 3 h. However, ETHO2R exhibited an exponentially increasing pattern of the drug permeation profile with the square root of time. This suggested the impact of ethanol content in ETHO2R for maximized solubility of the drug at the skin–air interface while the vesicle–skin interaction took place. In gel formulation, the content of water is responsible for diluting the ethanol of ethosomes, and the high viscosity slowed down vesicle–skin interaction. Theoretically, alcoholic vehicles are responsible for increasing the permeability of the drug permeation across the SC layer due to ethanol-mediated increased skin lipid fluidity within the intercellular spaces (30 nm) and extending the hydrophobic domain between polar head groups in the SC [27,28,29].

There are several factors affecting the permeation profile of lipophilic/hydrophilic drug trapped in vesicular systems, such as ethosomes and elastic liposomes. The improved permeation and diffusion of the drug across rat skin can be explained based on the composition (ethanol, lipid, and edge activator), skin condition, and hydration (gel). Ethanolic ethosomes cause variation in the phospholipid configuration of SC variation from steady state to unstable state due to decreased transition temperature (T_m_) and enthalpy of the treated SC (20–25 layers of dead cells in human) layer of the skin [30]. This transition indicates an increase in the fluidity of SC layer lipids that could facilitated vesicle permeation across the SC layer for dermal access. Thus, ethanol and phospholipid of ethosomes had a substantial impact on the fluidization and reversible disruption of skin lipid bilayer [30]. Ethyl alcohol (high content in ethosomes) increases the skin penetration of the drug due to increased solubilization in the gel form of ETHO2-GR and their combined effects resulted in high permeability coefficient and diffusion coefficient as compared to respective ELP3R (low content of ethanol ~ 7%) and ELP3-GR (Table 1). Comparing ethosomes with elastic liposomes, it was observed that ethosomes were remarkably more squeezable and permeable for enhanced permeation, as evidenced with permeation and diffusion parameters (Table 1). This can be correlated with relatively small sized ethosomes (128 nm), high drug %EE (98.1%), and more elasticity (63.2) due to high ethanol content. The high diffusion coefficient (0.104 × 10^−4^ cm^2^/h) of ETHO2R may be attributed to the higher rate of diffusion (0.315 mg/cm^2^/h^1/2^) of the drug through rat skin as a result of the higher concentration gradient developed by the components of ethosomes (improved solubilization).

Hansen solubility parameters of ACV are 19.4, 13.6, and 13.7, for δ_d_ (dispersion ability parameter), δ_p_ (polarity due to dielectric constant value), and δ_h_ (hydrogen bond formation ability parameter), respectively. The drug is associated with three hydrogen bond donor counts and five hydrogen bond acceptor counts. Pure ACV is poorly soluble in ethanol, whereas the sodium salt of the drug is soluble in water. Thus, ethosomes are capable of loading the drug in aqueous compartments (fraction of the drug available as salt) and lipid bilayers (fraction of drug free from salt). Hansen reported the values of δ_d_, δ_p_, and δ_h_ as 17.6, 12.6, and 11, respectively, for healthy human SC [31]. Abbott, 2012, first reported the values of δ_d_, δ_p_, and δ_h_ as 17, 8, and 8, respectively, for human SC in solvents to tailor optimized formulation as human skin represents its polarity due to the free functional groups of skin protein and polysaccharides. These parameters (δ_d_ = 17.6, δ_p_ = 12.6, and δ_h_ = 11) helped us to understand molecular mechanistic perspective of the drug interaction with skin composition and drug–vesicle interaction [32,33]. The lowest value of difference of any Hansen parameter between solute and solvent/polymer (considering skin as complex polymer) indicates maximum interaction or miscibility. This can also be correlated with a high number of hydrogen bond acceptor counts of the drug for maximized interaction with human skin. Thus, it is quite clear that the values of δ_d_, δ_p_, and δ_h_ for the drug and human skin (literature value) are closely related, which give the lowest differences (δ_p_ of ACV—δ_p_ of human skin = 1.8, and so on). Thus, ethosom entrapped drug was maximally permeated, diffused, and deposited within the dermal region after permeation across the SC layer. The lodged drug was slowly released within the skin matrix for extended release. Considering the impact of carbopol gel, the hydrophilic gel is a single-phase transparent matrix with a high content of water. After topical application, the resulting thin film is readily absorbed followed by vesicular adhesion with skin. Vesicles may interact to be pushed inward across the SC layer as a result of established hydration (the intrinsic transcutaneous hydration pressure difference) and concentration gradients [29]. Diffusion and permeation data obtained in the human skin would be the most relevant to establish an in vivo performance and mechanistic perspective. However, in vitro-ex vivo permeation and diffusion data generated using the EpiDerm membrane and rat skin are still valuable to investigate impact of ethosomes vesicle and gel on the permeation and diffusion rate from the topically applied site.

### 3.5. In Vivo Transepidermal Water Loss (TEWL) Study Using Rat Skin

The values of TEWL were estimated for A–D and compared against untreated control group E. These values were determined at zero time point and at the end of the study (3 h).

Generally, TEWL for healthy human skin (untreated) ranges from 0 to 10 g/m^2^h [27]. The evaporation of water (loss of water) from skin is measured as TEWL. Heavy water loss of the rat skin (aquaporin-1 and 3) was attributed to a poorly developed epidermis or formulation-treated skin [34]. The values of TEWL for group A, B, C, D, and E were found to be as 5.3, 5.2, 4.9, 4.7, and 6.1 g/m^2^h, respectively, at 0 h. These values indicate the normal nature of rat skin. These values for group A, B, C, D, and E were found to be as 14.7, 21.9, 10.2, 18.6, and 5.9 g/m^2^h, respectively, at 3 h. Considering these values obtained as real-time TEWL, it is easy to compare and explain the impact of ethosome, elastic liposomes, and gel formulation. The highest TEWL value was associated with ETHO2, which suggested the ethanol-mediated maximum interaction of ethosome vesicle with SC layer of epidermis, whereas its respective gel caused slightly decreased TEWL values due to gel-based hydration and ethanol dilution. However, these values are in good agreement with the published report for its normal architecture of rat skin [27,35]. These data obtained are informative to understand reversible perturbation imposed after topical application. In the case of elastic liposomes, the values of TEWL were relatively low (14.7 g/m^2^h for ELP3R and 10.2 g/m^2^h for ELP3-GR) as compared to ethosomes. It may be prudent to correlate the difference in ethanol content in the formulation.

### 3.6. Confocal Laser Scanning Microscopy (CLSM)

**Figure 3:**
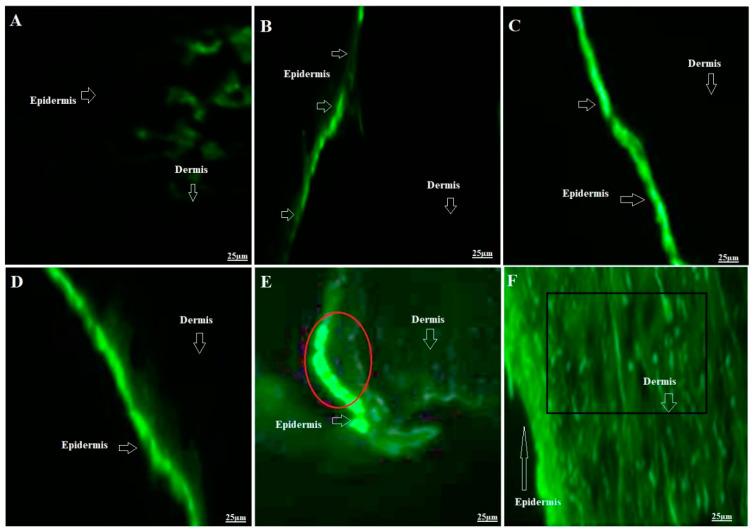
Confocal laser scanning microscopy of untreated control and treated rat skin. (**A**) Untreated rat skin exhibiting no intense fluorescence from epidermis SC layer and dermis region, (**B**) dye-treated (rhodamine aqueous solution) group exhibited poor fluorescence after topical application due to limited access across crystalline SC layer, (**C**) ELP3R-treated rat skin exhibiting intense fluorescence color due to facilitated permeation of elastic liposomes via intracellular squeezing and vesicular deformation, (**D**) ETHO2R-treated rat skin exhibiting intense fluorescence color due to the facilitated permeation of ethosomes across SC layer by ethanol-based skin lipid amalgamation and conformation changes (reversible changes) resulting in SC layer perturbation, (**E**) ELP3GR-treated rat skin exhibiting significant accumulation (indicated in red circle) of elastic liposomes in SC layer and subsequently drug delivery to the dermal area (as evidence with weak fluorescence in dermal region), and (**F**) ETHO2GR-treated rat skin revealing uniform and consistent delivery of ethosomes from epidermal region to dermal area, as evidenced with prominent fluorescence in the epidermis and dermal region (indicated in the black square).

After completion of ex vivo permeation and TEWL studies, it was imperative to visualize the epidermal changes after treatment. It was anticipated that the applied formulation would cause remarkable reversible epidermal changes (particularly SC layer) for augmented drug diffusion and permeation, as evidenced with permeation parameters (obtained). Therefore, CLSM was purposely employed to see the degree of vesicle penetration as intact or squeezed or in term of fluorescence intensity. To evaluate the mechanistic perspective of the developed binary vesicular system for permeation and penetration, human skin was the best candidate. However, rat skin was selected due to comparable structural similarity (in term of thickness), availability, and nearly similar permeation pharmacokinetics. Authors reported that the thickness values of SC layer, epidermis, and the whole skin are 17 µm, 47 µm, and 3 µm for humans, whereas these values for rats are 18 µm, 32 µm, and 2 µm, respectively [36,37]. Follicular permeation and rate of drug diffusion is relatively high in rats due to dense hairs density in rats compared to humans. The result is portrayed in Figure 3A–E. Figure 3A exhibited no permeation intensity across SC layer due to the untreated group, whereas the dye solution-treated group showed the accumulation of the dye on the SC layer with limited (or no permeation) permeation (Figure 3B). Figure 3C,D revealed remarkable and intense fluorescence intensity of ELP3R and ETHO2R, respectively, which may be correlated due to maximized internalization of vesicles with SC layer and ethanol-based extraction of skin lipid. These vesicles significantly interacted with the skin composition (skin lipid and matrix) and caused reversible changes to offer ways for vesicle (elastic liposomes and ethosomes) to be squeezed across tiny pores (generally reported pore size as 0.3 nm and opened without damage merely to 40 nm) and penetrated to the deeper region (dermal area) following tortuous intercellular spaces [38]. ETHO2R exhibited highly intense SC layer as compared to ELP3R which may be due to ethanol-mediated perturbation, surface lipid extraction, and reversible changes. Moreover, the ETHO2R-treated group elicited the structural changes in SC layer and relatively more diffusion of the probed vesicles across the SC layer as compared to ELP3R. Notably, both ELP3R and ETHO2R were aqueous vesicle suspensions (pH 7.4) and therefore vesicles were expected to be permeated across the SC layer slowly, and the excess water from the suspensions typically evaporated in ~0.5 h (forming a macroscopically dry lipid film), and it was believed to be a highly concentrated vesicle suspension thin layer left behind on epidermal side [38]. The mucoadhesive carbopol (pH ~5–6.8) exhibited the highest degree of penetration across SC layer, as shown in Figure 3E,F. ELP3GR showed maximum vesicle accumulation in the SC layer (red circled region in Figure 3E with intense fluorescence) followed by squeezed to dermal region which may be due to relatively larger vesicle size than ETHO2GR. ETHO2GR showed substantial penetration across SC layer with no accumulation within SC layer and maximum penetration to the dermal region (rectangle). Thus, carbopol-based gel-assisted ETHO2R and ELP3R for the augmented permeation across the SC layer, which may be correlated to the combined impact of TEWL (transepidermal water loss), ethanol-based skin lipid extraction, vesicle interaction (high deformability and ultradeformability), mucoadhesiveness, viscosity for high residence time, and surfactant-mediated interaction with skin composition.

### 3.7. Vesicle–Skin Interaction Study Using Scanning Electron Microscopy (SEM)

**Figure 4:**
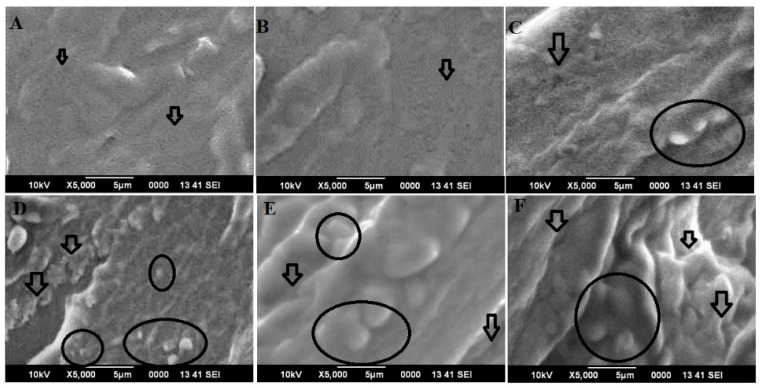
Representative images of scanning electron microscopy (SEM): (**A**) untreated rat skin with intact surface without significant changes, (**B**) the dye treated rat skin with similar morphology and surface topology, (**C**) ELP2R elicited skin surface perturbation higher than the control (untreated) (as indicated in black circle), (**D**) ETHO2R executed relatively higher skin surface perturbation (as indicated in black circle) as compared to controls (untreated and the dye treated) and ELP2R, (**E**) ELP2GR exhibited higher skin perturbation higher than respective ELP2R colloidal suspension which may be attributed to gel mediated hydration effect (as indicated in black circle), and (**F**) ETHO2GR exhibited higher skin perturbation higher than respective ETHO2R colloidal suspension which may be attributed to gel and high content of ethanol mediated perturbation (as indicated in black circle).

The skin tissue specimens obtained from an ex vivo study were used for visualization under SEM. The technique helped to examine microscopic changes in the treated skin surface and permeation of vesicle across rat skin. The dye (rhodamine 123) is a cationic dye and cell permeant giving green fluorescence without cytotoxicity. The dye is hydrophobic with limited aqueous solubility, comparable molecular weight to the drug, and is commonly used as dye because of intense fluorescence and the stable green emission of fluorescence at the described wavelength. Moreover, the dye is a substitute of the drug to visualize the permeation and penetration of the lipophilic vesicle and the drug distribution across rat skin due to shared common characteristics such as reasonable molecular weight (225 g/mole of ACV versus 380 g/mole of the dye), hydrogen bonding donor counts (2 versus 2), hydrogen bonding acceptor counts (7 versus 5), and high lipophilicity (*p* ˃ 1.5) [39]. To visualize the surface morphology of untreated rat skin and treated skin, SEM showed apparent surface view while scanning, as shown in Figure 4A–F. It was found that untreated rat skin exhibited an intact skin surface with no visible changes or microscopic perturbation (fissures) and a similar pattern of morphology was observed with the skin treated with dye solution (Figure 4A,B). This may be due to insoluble aqueous dye being unable to interact with the SC lipid and composition (Figure 4B). In the case of vesicular formulations, the result was quite convincing, as we hypothesized. The colloidal suspensions of ELP2R and ETHO2R were maximally interacting with the skin surface for squeezing through microscopic skin pores and follicular pathways (Figure 4C,D) [20]. ETHO2R executed relatively higher skin surface perturbation (as indicated in black circle), reversible changes, and penetration, which may be attributed to the high content of ethanol (~45%) and small vesicles, as shown in Figure 4D [20]. This was a supportive finding to confirm the behavior difference between ethosome-based vesicle suspension (ETHO2R) and respective gel (ETHO2GR). In an ex vivo study (Section 3) it was explained that ETHO2GR showed slow and sustained permeation parameters, whereas ETHO2R revealed a remarkably high permeation flux. This was explained based on the fact that high flux is appreciable only when significant size fragmentation occurs while passing across tiny, microscopic pathways of skin strata, whereas intact vesicles pass through the tortuous pathways at a slow rate of permeation [14,38]. Moreover, there are various extrinsic and intrinsic factors controlling permeation rate, drug diffusion, and penetration profile. These are physicochemical properties of the drug, excipients and formulation, physiological content of skin (normal or diseased), method of delivery (occlusive or non-occlusive), pharmaceutical parameters (dose, dosage form, and dosing frequency), and environmental condition (geographic and race variation). Conclusively, ETHO2 and respective gel caused maximum skin perturbation and surficial reversible changes as compared to others for enhanced transdermal drug delivery (Figure 4E,F).

### 3.8. Atomic Force Microscopy (AFM) Study

**Figure 5:**
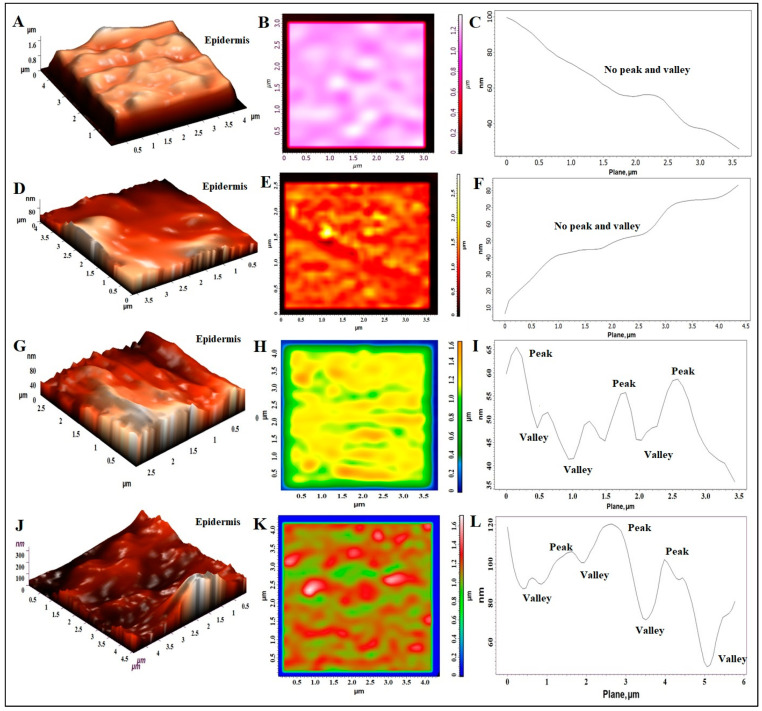
Topological study of untreated and treated rat skin using atomic force microscopy (AFM). (**A**) Three-dimensional image of control rat skin (untreated) exhibiting natural surface morphology of stratum corneum (SC) of epidermis, (**B**) two-dimensional image of untreated rat skin revealing surface margin and layering with intact SC layer without gaps and perturbation, (**C**) surface roughness curve profile of untreated skin with no prominent peak and valley indicating intact skin, (**D**–**F**) 3-D, 2-D, and surface roughness curve parameter of dye-treated rat skin without prominent peaks and valley (comparable to untreated control), (**G**–**I**) 3-D, 2-D, and surface roughness curve parameter of ELP3R-treated rat skin with prominent peaks and valley indicating reversible structural changes in SC surface, and (**J**–**L**) 3-D, 2-D, and surface roughness curve parameter of ETHO2R-treated rat skin with prominent peaks and valley indicating reversible structural changes in SC surface. ETHO2R caused relatively more roughness on SC surface, as evidenced with amalgamated skin lipid (3-D image (**J**)), and created microscopic gaps (2-D image in (**K**)).

There are various factors that are responsible for causing reversible changes in the skin architecture after topical application. These are compositions (lipid and surfactants), gel-based hydration, and the physiological condition of skin. To support our assumption and SEM findings, AFM was imperative to perform the surface roughness assessment. The result is portrayed in Figure 5A–L. The technique provided various topological parameters such as (a) average roughness (Ra, nm), (b) root mean square as “rms” in nm, (c) surface skewness (is a measure of the symmetry of the height distribution and the number of hills and valleys on the surface), and (d) entropy (degree of disorderness). The coefficient of Kurtosis (Rka) measures the distribution sharpness and highlights the peak or valley profile in terms of steepness or roundness (whether peaks and valleys are steep or round). Figure 5A–C indicted a three-dimensional topographic view, two-dimensional AFM image, and surface roughness curve profile of untreated rat skin, respectively. 3-D and 2-D images confirmed the roughness of the skin due to corneocytes disposition typical in healthy skin, which was further supported in the roughness curve with no prominent peaks and valleys (Figure 5C) [40]. AFM analysis reported that the values of Ra, rms, surface skewness, and entropy of untreated rat skin (25 µm × 25 µm) were found to be 33.47 nm, 49.95 nm, 1.86, and 10.25 J/K, respectively. The untreated skin showed a smooth surface with no gaps or intercellular spaces. A brighter zone was preferred over the darker area (intercellular gaps) to avoid any border effect while scanning with cantilever. The two-dimensional image showed no significant gaps and fissure on the surface (Figure 5B). Figure 5D–F is the dye-treated rat skin which exhibited no substantial perturbation and surface roughness compared to untreated control skin. Considering the analysis report, the values of Ra, rms, surface skewness, and entropy were found to be 15.90 nm, 55.84 nm, 1.27, and 5.79 J/K, respectively, which are close to untreated skin. The slight difference in these values may be attributed to water-mediated swelling of the skin surface. However, the two-dimensional image showed no significant gaps (intercellular spaces) and the roughness curve profile was observed in a similar pattern in term of peaks and valleys. ELP3R and ETHO2R elicited remarkable changes in surface topographic pattern, as evidenced with Figure 5G–I and Figure 5J–L, respectively. It is apparent from the surface analysis report that AFM assessed different surface patterns which may be correlated to the composition of elastic liposomes (7% ethanol as plasticizer) and ethosomes (containing 45% ethanol). Despite these, other factors play a considerable role for topographical changes for improved drug permeation and penetration. The values of Ra, rms, surface skewness, and entropy were found to be 8.99 nm, 72.42 nm, −0.6, and 14.5 J/K, respectively, for ELP3R-treated rat skin. The negative of the surface skewness characterized the surface with deep voids or cracks (microscopic fissures), and this can be confirmed by the gaps clearly observed in Figure 5H. A similar observation was inspected in Figure 5K due to ethosomes-mediated surface perturbation. Looking at the surface roughness measured parameters, the values of Ra, rms, surface skewness, and entropy were found to be 95.68 nm, 240.91 nm, −0.98, and 29.04 J/K, respectively, for ETHO2R-treated rat skin. A higher negative value (−0.98 ˃ −0.6) of surface skewness indicates relatively deeper voids or cracks generated after topical application, which may be attributed to ethanol-mediated skin lipid extraction and reversible changes. In untreated and dye-treated controls, the positive roughness skewness could be explained by the lack of surficial perturbation, subsurface damage, and the absence of cracks or fissures propagating below the surface [41]. Skewness can be considered as reliable and a real change assessment parameter as compared to average roughness value. In certain cases, roughness values (Ra) may be similar, even in two different surfaces [42]. The values of coefficients of Kurtosis were estimated as 8.87, 0.76, −1.19, and −1.4 for untreated, dye-treated, ELP3R-treated, and ETHO2R-treated groups, respectively. The higher Kurtosis value of untreated skin was found due to round and short peaks with no deep voids and cracks, whereas a more negative value of Kurtosis in the skin treated with ETHO2R could be associated with apparent voids, cracks, and deep figures generated after treatment. Thus, mean roughness, skewness, and Kurtosis parameters were the most reliable and useful topographical parameters to explain the surface roughness of rat skin [42].

## 4. Conclusions

Based on the findings, it was imperative to conclude the impact of the optimized ETHO2 and ELP3 on permeation behavior across three different barriers. Moreover, a mechanistic perspective of permeation and penetration across rat skin and EpiDerm was elaborated using advanced techniques such as CLSM, SEM, and AFM. The reformulated ETHO2 and ELP3 (containing rhodamine 123) and their respective gels were studied to understand the mechanistic insight of the permeation and penetration of the two selected vesicular systems. We highlighted the relative findings of both reformulated products for improved therapeutic benefits. It is noticeable that the values of vesicle size difference were found to be insignificant, whereas the respective gel showed a slight increment in vesicle size of both products due to the hydration effect of gel. The high content of ethosomes ETHO2R had a great impact on the vesicle size, permeation profiles (flux, permeation coefficient, diffusion coefficient, and diffusion rate), and penetration compared to ELPR3R. The TEWL study suggested that ETHO2R reduced a significant amount of skin water, which may be attributed to ethanol-mediated water extraction. However, gel formulation (hydration effect of gel) reduced TEWL loss relatively lower than the respective ETHO2R. The surface perturbation was visualized under SEM and CLSM to confirm high drug accumulation within skin using ETHO2GR. AFM supported SEM and CLSM findings in terms of nanoscale surface topographical parameters. Thus, ethosomes-based ACV delivery is promising to control cutaneous herpes and herpes keratitis.

## Figures and Tables

**Table 1:** Composition and evaluated parameters of rhodamine-123 probed binary ethosomes and elastic liposomes and their respective gels for topical delivery of 5% *w*/*w* ACV gel (in 0.5% gel).

Code	PC:S	Chol (mM)	PG/Et (%)	R123 (%)	Size (nm)	η (cP)	%EE	E	pH
ELP3-R	85:15	-	-	0.1	217 ± 15	4382 ± 105	85.2	46.8	7.0
ETHO2-R	60:0	25	0.5	0.1	128 ± 19	4818 ± 117	98.1	63.2	7.1
ELP3-gel-R	85:15	-	-	0.1	231 ± 13	5983 ± 132	-	-	6.8
ETHO2-gel-R	60:0	12.5	0.25	0.1	252 ± 32	5872 ± 152	-	-	6.9

Note: E = Elasticity, PC:S = Phosphatidylcholine: surfactant, Chol: Cholesterol, PG/Et: Propylene glycol/ethanol, R123: Rhodamine 123, %EE = Entrapment efficiency.

**Table 2:** Summary of permeation and diffusion coefficients, flux, and correlation coefficients values of ACV obtained from the release plot of ELP3R, ETHO2R, ELP3-GR, and ETHO2-GR across the synthetic membrane, EpiDerm, and abdominal rat skin.

Code	Flux (µg/cm^2^/h)	* D (cm^2^/h)	* *p* (cm/h)	Diffusion Rate (mg/cm^2^/h^1/2^)	r^2^
Synthetic Membrane					
ELP3R	37.42	7.98 × 10^−6^	0.32 × 10^−3^	0.0648	0.761
ETHO2R	234.94	7.57 × 10^−5^	0.45 × 10^−3^	0.406	0.810
ELP3-GR	43.44	2.52 × 10^−5^	0.42 × 10^−3^	0.0752	0.845
ETHO2-GR	78.42	8.24 × 10^−5^	0.67 × 10^−3^	0.135	0.836
	Human cultured skin EpiDerm				
ELP3R	28.61	4.73 × 10^−6^	0.25 × 10^−3^	0.049	0.722
ETHO2R	169.58	3.51 × 10^−5^	0.63 × 10^−3^	0.293	0.865
ELP3-GR	88.55	1.05 × 10^−4^	0.36 × 10^−3^	0.153	0.87
ETHO2-GR	136.45	2.4 × 10^−4^	0.80 × 10^−3^	0.236	0.844
	Rat abdominal skin				
ELP3R	70.03	3.54 × 10^−5^	0.126 × 10^−2^	0.121	0.763
ETHO2R	182.42	1.05 × 10^−4^	0.3 × 10^−2^	0.315	0.744
ELP3-GR	65.55	5.75 × 10^−5^	0.11 × 10^−3^	0.113	0.869
ETHO2-GR	88.64	1.64 × 10^−4^	0.24 × 10^−3^	0.153	0.874

* Note: P and D indicate permeability and diffusion coefficients, respectively. The r^2^ represents the correlation coefficient of plot for cumulative drug released versus the square root of time (h). Data are mean value, taking *n* = 3 and *p* ˂ 0.05 as significant.

## Data Availability

Not applicable.
